# The impact of maternal and child health and nutrition improvement project on maternal health service utilization in Ghana: An Interrupted time series analysis

**DOI:** 10.1371/journal.pgph.0000372

**Published:** 2022-04-26

**Authors:** Arif Mohammed, Duah Dwomoh, Justice Nonvignon

**Affiliations:** 1 Department of Health Policy, Planning and Management, School of Public Health, University of Ghana, Legon, Ghana; 2 Department of Biostatistics, School of Public Health, University of Ghana, Legon, Ghana; African Population and Health Research Center, KENYA

## Abstract

Maternal and Child Health and Nutrition Improvement Project (MCHNP) is an intervention that, adopts financial strategies to provide incentives as a means of motivating community health workers and ensuring accountability. This study highlights on the service delivery component of the intervention; thus, utilization of essential community nutrition and health action. This paper aims to determine the differential impact of MCHNP on maternal health service utilization in Ghana. A retrospective longitudinal pre-test post-test study design was employed. Six administrative regions were used for analyzing the impact of the intervention in uptake of maternal health services. Administrative data were extracted from the DHIMS2 database for the periods of January 2014 to December 2018. Analysis was conducted using interrupted time series analysis (ITSA) due to the absence of a control group. The difference in the pre-intervention and post-intervention means were statistically significant in the Central, Western, Eastern and Upper West region for the proportion of ANC 4 visits. With the exception of Northern region that recorded negative impact (-0.005; p-value >0.05), all the remaining regions recorded positive impacts on the percentage of women that had 4 ANC visits. All six regions had positive impacts in the proportion of women that received supervised delivery. However, none of these impacts were statistically significant; thus, the MCHNP intervention had no significant impact on maternal health outcomes which are, ANC four visits and skilled deliveries.

## Introduction

Maternal and child health continue to receive major attention on the global health landscape as evidenced in major global goals such as the Sustainable Development Goals (SDG) [1]. A joint report by the World Health Organization (WHO) and United Nations Children’s Fund (UNICEF) in 2014, revealed an improvement in maternal health services utilization, with about 86% of pregnant women accessing prenatal care globally [2, 3]. Studies have however indicated that, Antenatal Care (ANC) and delivery care are amongst the vital components of an improved maternal health care package [4]. Focused ANC came into being following its promotion by the WHO where every pregnant woman visit a health facility for at least four times in the case of an uncomplicated pregnancy [5]. Recent clinical studies have indicated improved health outcomes, should one ascribe to the recommended ANC visits [6]. Following this assertion, it is indicated that irregular attendance for antenatal care predisposes one to pregnancy complications such as pre-eclampsia, eclampsia and anemia beside adverse birth outcomes including preterm birth, low birth weight and stillbirth [6]. UNICEF and other studies have reported that only 60% of women worldwide had at least four ANC visits, and only about 52% of women from sub-Saharan Africa (SSA) countries had at least four ANC visits during the period of their pregnancy [7]. Other studies also presented similar findings by indicating that, 50% of women in SSA were able to receive the WHO recommended four ANC attendance [8] and 58% of children were delivered by a skilled birth attendant [9].

### Intervention

The Maternal and Child Health and Nutrition Improvement Project (MCHNP) is a performance-based financing (PBF) initiative funded by the World Bank (WB), that sought to improve health and nutrition outcomes, amongst women of reproductive age and children aged less than 2 years [10–13]. As a PBF, MCHNP incorporates financial incentives for Community Health Workers (CHWs) [10]. Key to this intervention is adapting to financing strategies to improve incentives for CHWs, increase availability, accessibility, affordability and ensuring accountability. MCHNP intends to expand geographical coverage of essential services (antenatal care and delivery, counseling of new mothers, management of childhood illness at home, growth monitoring). This is one of the components of the intervention. The other component seeks to focus on institutional capacity building. This component tends to strengthen health systems to improve the quality and quantity of services provided, by improving uptake and accountability, as well as improving access and affordability [10].

The intervention was initially piloted in eight districts in four resource constraint regions in Ghana before its nationwide rollout in the 2014. It was a five-year project whose implementation spanned from 2014 to the year 2018. In summary, the MCHNP intervention encompasses community-based maternal and child health interventions and also community-based nutrition interventions.

PBF initiatives in recent times however, have received several critiques following the Cochrane collaboration systematic review on PBF in 2012, which indicates that, PBF in Low and Middle-income Countries (LMICs) are based on too weak evidence and very little experience for a large scale implementation, hence the tendency to weaken the existing health system [14, 15]. The objective of this study, therefore, was to determine the differential impact of MCHNP on maternal health service utilization (ANC four visits and supervised delivery) in Ghana.

## Method

### Study design

The study employed a retrospective pre and post-test study design, which is a longitudinal study design. Monthly data on outcome variables were collected from January 2014 to December 2018. Aggregated measurements were obtained for the outcome variables on monthly basis for the target population for all the regions of Ghana. The selection of this design helped to measure effects of the intervention over time on the outcomes of interest. As the strongest quasi-experimental design, a total of 60 data points/observations were produced to accurately estimate the effect of the intervention. The adoption of interrupted time series design was as a result of the lack of a control group, given that the intervention was implemented nationwide.

### Study setting

The study was conducted in six (6) administrative regions of Ghana namely; Central, Eastern, Northern, Upper East, Upper West and Western regions. The geographical location of these regions predisposes residents to diverse health seeking behaviors. The Northern, Upper East and Upper West regions can be found in the northern part of the country. The Central and Western regions are placed in the coastal belt, with Eastern region residing in the middle belt of the country. Generally, the health system is challenged with inadequate staff in the northern part of the country due to the remote nature and low living standards in most of its districts. The north is also challenged with poor health seeking behaviors, as most of the resident originated from traditional homes with low educational levels compared to their counterparts in the coastal and middle belt of the country. Communities in the north are sparsely dispersed making access to available health services difficult in the regions.

The middle and coastal belts though encounter some of the challenges in the north, but are a bit better off. Regions in these zones are mostly cities where commercial activities are predominant. Cost of living in most of these areas are standardized and residents with most residents’ having received higher educations. Health seeking behavior in these areas are quite different from that of residents in the north.

### Study population

The study population were women of reproductive age (10–49 years) over a period of five (5) years as captured in District Health Information Management System 2 (DHIMS2). Therefore, pregnant women who accessed antenatal service and women who accessed delivery services in the health facilities were used. Intervention sites were health facilities across the country. The unit of analysis for this study however was the regions. In addition, the six (6) regions have an estimated total women population of 7.4 million.

### Sample size

A total sample of 4,713,051 women of reproductive age was used for the study, from the six (6) selected regions.

### Inclusion criteria

All regions with health facilities providing maternal health care services and were reporting in DHIMS2 from January 2014 to December 2018. The study included regions whose data were verified and certified/ signed off by their Regional Health Information Officers (RHIOs).

### Exclusion criteria

The exclusion criteria for the study were regions who had health facilities providing maternal health services from January 2014 to December 2018, but whose RHIOs officer could not sign off the data for use, due to data inconsistencies.

### Data collection

Data for the study were obtained from the national health database viz; DHIMS2. The database is a web-based system that collects both transactional and aggregated data. Data and or indicators for this study thus; number of pregnant women who had four ANC visits, as well as those who received skilled delivery forms part of the aggregated data captured by the system. These data are collected and entered into the system by designated officers at the service delivery points (health facilities). Service data from January 2014 to December 2018, were extracted from the system to enable the researchers to assess the impact of the intervention on maternal health services for the study period for the six (6) selected regions. The retrieved data from the DHIMS2 database were downloaded into an excel format to enhance data validation, cleaning and other management.

### Data management

To ensure high quality data, the data extracted were sent to RHIOs to cross check with what their respective facilities have submitted and entered into the system. Another line of validation which the researchers also used was to download other datasets in the system (DHIMS2), which also reports on the outcomes of interest. This was done because some data elements are cross cutting on other datasets and uses that as a proxy for the validation.

Incomplete or missing information were present when the data was first extracted. The issues were identified and follow up visits were made to the various regions for completion of missing data points and elements. The RHIOs consulted their district and facility HIO for rectification using the source register, before finally signing off the data as the true and certified copy for the study. In other words, RHIO were tasked to correct any data anomalies and inconsistencies and duly effect the changes on DHIMS2. At the end of the validation process, six regions had completely cleaned and reconciled the inconsistencies. The four other regional officers could not work within the allocated space for the data validation and hence were excluded from the analysis. To avoid data loss in the future, a copy of the validated data was kept in cloud storage to ensure future accessibility.

### Indicators

The study outcome indicators were the proportion of pregnant women who had four antenatal visits and also the proportion of women who received supervised delivery by a qualified professional over the study periods. ***Table 1*** below presents the details on the outcome indicators.

**Table 1:** Description of outcome variables.

Indicator	Description	Scale of Measurement	Measurement
Antenatal four visits	Number of pregnant women who made four visits to the antenatal clinic for check-up	Discrete	Count
Skilled delivery	Number of pregnant women who were delivered in a certified facility by qualified professionals	Discrete	Count

### Statistical analysis

Background information on maternal health service utilization was summarized and further segregated into pre-intervention summary (January 2014—December 2015) and post-intervention summary (January 2016 –December 2018).

An interrupted time series analysis (ITSA) regression model was employed to determine the differential impact of MCHNP on the maternal health service utilization, using the underlying trends determined over the study period. The adoption of ITSA was because it is the strongest quasi-experimental design considering the data available (data before and after intervention). To reiterate, ITSA was employed as a result of lack of a control group, given the fact that the implementation of the intervention was nationwide. Also ITSA offers a hypothetically high level internal validity given that the intervention had no comparison group [13]. In effect, the study used ITSA due to the lack of randomization.

Time series data are said to encounter serial correlation or autocorrelation as well as heteroscedasticity in the model’s error term. Cumby-Huizinga test for autocorrelation was employed to overcome serial correlation and heteroscedasticity at specified lags as presented in S1 Table.

The analysis also controlled for other interventions which had occurred concurrently with the evaluated intervention. These interventions (confounding factors) were, Millennium Development Goal (MDG) Accelerated Framework (MAF) and MEBCI (Making Every Baby Count Initiative) which all sought to improve maternal and child health services and outcomes in the jurisdiction.

### Estimation technique

ITSA relies on ordinary least square (OLS) regression, due to its flexibility in context and its ability to account for autocorrelated errors [16].

A single-group ITSA must be employed when the study has no comparison group. that is, using only one group [11, 17]. This makes the adoption of single group ITSA suitable for the estimation of the differentiated impact of maternal health service utilization and expressed mathematically as;

$$Y=\beta_{0}+\beta_{1}T+\beta_{2}X+\beta_{3}TX+\varepsilon$$

Where; **Y** is the outcome variable of interest measured, **T** is the time periods specified for the study, ***X*** is a dummy variable representing the intervention (pre-intervention periods 0, post-intervention 1), **T*X*** is an interaction term and represent the impact of the intervention and ***ε*** been the error term.

In the case of a single-group study, *β*_0_ represents the intercept or starting level of the outcome variable. *β*_1_ is the slope or trajectory of the outcome variable until the introduction of the intervention. *β*_2_ represents the change in the level of the outcome that occurs in the period immediately following the introduction of the intervention (compared with the counterfactual). *β*_3_ represents the difference between pre-intervention and post-intervention slopes of the outcome variables” [16].

Statistical analysis was conducted using Stata IC 15.0 (StataCorp, College Station, USA).

### Ethical statement

No ethical clearance was needed for this study. Data used for the study was a population data and cannot be attributed to an individual participant. However, permission was sought through the University of Ghana, School of Public Health to the Director Policy, Planning, Monitoring and Evaluation Division (PPMED) of the Ghana Health Service (GHS), to use the data from DHIMS2 to evaluate the impact of this important intervention.

## Results

### Background characteristics of maternal health service utilization

***Table 2*** displays the utilization of maternal health services for the pre-intervention and post-intervention periods. ANC registrants saw an increase from 40.41% in the pre-intervention era compared to a 59.59% post-intervention. Deliveries increased substantially from 1,243,769 (38.50%) to 1,986,433 (61.50%) pre-intervention and post-intervention respectively.

**Table 2:** Background characteristics of maternal health services.

Variables	Pre-intervention (n)	Percentage (%)	Post-intervention (n)	Percentage (%)	Total (n)
**ANC Registrants**					
10–14	5,987	44.03	7,611	55.97	13,598
15–19	225,109	40.71	327,791	59.29	552,900
20–24	487,548	41.68	682,292	58.32	1,169,840
25–29	540,034	40.72	786,080	59.28	1,326,114
30–34	400,264	39.48	613,506	60.52	1,013,770
35 and above	245,401	38.53	391,428	61.47	636,829
**Total**	**1,904,343**	**40.41**	**2,808,708**	**59.59**	**4,713,051**
**Trimester**	** **	** **	** **	** **	** **
1st trimester	862,623	39.38	1,328,024	60.62	2,190,647
2nd trimester	784,834	40.67	1,144,749	59.33	1,929,583
3rd trimester	238,087	42.67	319,901	57.33	557,988
**Total**	**1,885,544**	**40.30**	**2,792,674**	**59.70**	**4,678,218**
**Parity**	** **	** **	** **	** **	** **
0	525,081	40.38	775,416	59.62	1,300,497
1–2	751,488	40.01	1,126,637	59.99	1,878,125
3–4	433,342	40.56	635,121	59.44	1,068,463
5+	186,290	41.69	260,532	58.31	446,822
**Total**	**1,896,201**	**40.40**	**2,797,706**	**59.60**	**4,693,907**
**Delivery**	** **	** **	** **	** **	** **
10–14	3,387	40.88	4,899	59.12	8,286
15–19	140,902	38.70	223,142	61.30	364,044
20–24	305,370	39.82	461,535	60.18	766,905
25–29	354,105	38.67	561,651	61.33	915,756
30–34	270,404	37.71	446,691	62.29	717,095
35+	169,601	37.02	288,515	62.98	458,116
**Total**	**1,243,769**	**38.50**	**1,986,433**	**61.50**	**3,230,202**
**Type of Delivery**	** **	** **	** **	** **	** **
Normal	1,056,569	39.14	1,642,809	60.86	2,699,378
Caesarean section	187,200	35.27	343,624	64.73	530,824
**Total**	**1,243,769**	**38.50**	**1,986,433**	**61.50**	**3,230,202**

### T-test comparing pre-intervention and post-intervention means of outcome variables by regions

***Table 3*** shows results of mean pre-intervention and post-intervention of maternal health service utilization, using unequal Welch t-test in the respective regions. Pre-intervention and post-intervention mean of ANC four visits was statistically significant (p-value <0.05) in the Central, Western, Northern and Upper West regions.

**Table 3:** Pre-intervention and post-intervention means of maternal health service utilization by regions.

Variables	ANC4 visit		Skilled Delivery	
	Pre-intervention	Post intervention	Pre-intervention	Post intervention
**Central**				
Mean	4.05	3.73	1.27	1.29
P-value	<0.01		0.55	
**Western**				
Mean	3.31	3.13	1.00	1.10
P-value	0.04		<0.001	
**Eastern **				
Mean	2.63	2.633	0.99	1.02
P-value	0.97		0.25	
**Northern**				
Mean	6.51	7.57	1.53	1.80
P-value	<0.01		<0.001	
**Upper East**				
Mean	2.79	2.70	0.98	1.08
P-value	0.49		0.05	
**Upper West**				
Mean	3.44	4.35	1.26	1.42
P-value	<0.001		<0.01	

**(1) Pre-intervention**: January 2014 –December 2015; (2) **Post-intervention**: January 2016 –December 2018.

The Western, Northern, and Upper West regions recorded a statistically significant (p-value <0.05) pre-intervention and post-intervention means for the percentage of women who received skilled delivery.

### Impact of MCHNP on the percentage of women who had four (4) ANC visits using interrupted time series analysis

***Table* 4** below presents impact estimate of maternal health service utilization thus, percentage of women who had four (4) ANC visits and percentage of women who received skilled delivery.

**Table 4:** Impact of MCHNP on service utilization using interrupted time series analysis.

Variables	Central Region	Western Region	Northern Region	Eastern Region	Upper East Region	Upper West Region
**Percentage of women who had four (4) ANC visits**						
**Time**	-0.007*	-0.006*	0.010	-0.007**	-0.01	-0.005
**Intervention**	0.057	0.076	0.192	0.175**	0.08	0.276
**Interaction/Impact**	0.004	0.003	-0.005	0.002	0.011	0.007
**Percentage of women who had skilled delivery**						
**Time**	-0.003	0.002	0.004	-0.005	0.001	0.001
**Intervention**	0.046	0.039	0.096	0.077	-0.058	0.059
**Interaction/Impact**	0.003	0.001	0.002	0.006	0.007	0.005

Significance level

*** p< 0.001

**p< 0.01

*p< 0.05; **Time**: Prior to the introduction of the intervention (January 2014 –December 2015); **Intervention**: First year of the intervention (January 2016); **Interaction / Impact**: Post-trend change (January 2014 –December 2018).

Northern region was the only region that had a negative post-intervention estimate (-0.05) in the percentage of women that had four ANC visits. There was an increased impact in the percentage of women had four ANC visits in the Central, Western, Eastern, Upper East, and Upper West regions with estimated impact of 0.004, 0.003, 0.002, 0.011, and 0.007 respectively. However, none of these impacts was statistically significant.

All the six regions recorded positive post-intervention estimates on the percentage of women who had skilled delivery. The Upper East and Eastern regions recorded the highest post-intervention estimates of 0.007 and 0.006 respectively. The Western and Northern regions on the other hand recorded the least post-intervention estimates of 0.001 and 0.002. These impacts though positive but was not statistically significant. The least post-intervention estimates recorded in the Northern and Western regions could be due to the long distances’ clients have to travel to nearby facilities for childbirth. The bad road network could also be a reason why these regions recorded marginal estimates as compared to their counterparts in the Eastern and Upper West regions.

## Discussion

The objective of the study was to estimate the differential impact of the nationwide implementation of MCHNP in Ghana on maternal health service utilization. The study found that the MCHNP intervention had positive impact on ANC in Central, Western, Eastern and Upper West regions of the country and on supervised delivery in all six regions evaluated. However, none of the impacts were statistically significant.

Uptake of ANC, thus the recommended four (4) antenatal visits during pregnancy saw positive impacts in the Central, Western, Eastern, Upper East and Upper West regions respectively, with the exception of Northern region which recorded negative impact; this could be due to late reporting at the ANC clinic. Late registration for prenatal care in the Northern mostly likely due to the health seeking behavior of the residents, where husbands and compound heads are known to gate-keep health service utilization by women [18]. None of the post-intervention impact estimates was statistically significant. This finding is consistent with findings from Burundi which concluded that, no evidence was found that PBF affected the likelihood of attending ANC [11]. The findings from this study were also consistent with impact estimates in Cameroon [19]. This notwithstanding, PBF incentives improved coverage and utilization of ANC and other maternal care services in Burkina Faso [20].

However, recent recommendations by the American Statistical Association argue that scientific conclusions, policy or business decisions need not be only based on whether p-value passes a specific threshold but also on clinical, social or policy significance [21].

Post-trend analysis revealed that the Central, Western, Eastern, Northern, Upper East and Upper West regions recorded positive impact on the percentage who received skilled or had facility delivery, however none of these positive impacts recorded was significant statistically. A study in Cameroun, indicated an absence of impact in skilled deliveries, which is similar to the impact recorded in skilled deliveries in all the six regions [19].

The statistically insignificant impact of MCHNP intervention on skilled delivery contradicts findings in Bangladesh, and Rwanda performance-based incentives significantly increased skilled delivery [11, 17]. The marginal, statistically insignificant impact from this study could be due to the fact that supply-side of the incentives by the providers were not enough given the prevailing health system and its challenges such as user fees and or unauthorized service charges [19]. Among other factors that could contribute to the insignificant impacts recorded are; poor design of the intervention to suit one’s local setting as the one fit all approach does not always work well in many settings. Again, limited availability of resources such as vehicles, logistics and staff, and also the late registration of pregnant women to the clinic later than the WHO recommend first ANC visit of twelve weeks of pregnancy could have contributed to insignificant impacts. Again, the cost transportation on the part of the client to the facility could be a reason to the insignificant impact.

The findings of this study imply that context is an important consideration in introducing financial incentive packages into a system as intricate as the health sector of countries to improve coverage and or performance [22]. It is, therefore, incumbent on program managers to combine demand-side and supply-side incentive to ensure effective utilization of the intervention. Efforts must be geared towards mechanisms that incentivize and improve the content of care beyond equipment and supplies [19]. Again, a review of the intervention’s design and other technical components as well as processes require review to determine how similar interventions could be implemented in future.

An important limitation of this study is that due to data incompleteness and inconsistencies in some of the regions, the study included data from six out of the ten administrative regions of Ghana.

## Conclusion

The implementation of MCHNP intervention had positive impacts on maternal health service utilization. With the exception of Northern region that recorded a negative impact in ANC 4 visits, the indicator saw a positive post-intervention estimate in the other five regions. The regions saw a positive impact estimates in the proportion of women who received skilled deliveries. All six regions used in the analysis of the intervention recorded positive impacts. However, none of these recorded impacts were significant statistically. Generally, the impact of the intervention was positive on maternal health services just that the impacts were not too high in the post-intervention evaluation.

## Supporting information

S1 Table Autocorrelation test for ITSA using Cumby-Huizinga test by regions. (DOCX)

S1 Data Extracted data. (XLSX)
