# Supplementary material for: The impact of maternal and child health and nutrition improvement project on maternal health service utilization in Ghana: An Interrupted time series analysis
Source: PLOS Glob Public Health. 2022 Apr 26;2(4):e0000372. doi: 10.1371/journal.pgph.0000372 (PMC10021576; doi:10.1371/journal.pgph.0000372)
Supplement: S1 Table — (DOCX) [file pgph.0000372.s001.docx]

**S1_Table: Autocorrelation Test for ITSA using Cumby-Huizinga Test by Regions**

| **Region** | **Variable** | **Lag** | **Chi square (χ2)** | **P-value** |
| --- | --- | --- | --- | --- |
| Central | Percentage of women who had four ANC visits | 1 | 5.12 | 0.024 |
|  | Percentage of women who had skilled delivery | 1 | 14.23 | <0.001 |
| Western | Percentage of women who had four ANC visits | 1 | 10.30 | 0.001 |
|  | Percentage of women who had skilled delivery | 1 | 11.27 | 0.001 |
|  | Percentage of women who had skilled delivery | 3 | 6.41 | 0.011 |
|  | Percentage of women who had skilled delivery | 1 | 6.85 | 0.009 |
| Eastern | Percentage of women who had four ANC visits | 1 | 6.89 | 0.009 |
|  | Percentage of women who had skilled delivery | 1 | 8.58 | 0.003 |
| Northern | Percentage of women who had four ANC visits | 1 | 27.80 | <0.001 |
|  | Percentage of women who had four ANC visits | 5 | 7.57 | 0.006 |
|  | Percentage of women who had four ANC visits | 6 | 10.01 | 0.002 |
|  | Percentage of women who had four ANC visits | 7 | 6.05 | 0.014 |
|  | Percentage of women who had skilled delivery | 1 | 24.13 | <0.001 |
|  | Percentage of women who had skilled delivery | 4 | 4.78 | 0.029 |
| Upper East | Percentage of women who had four ANC visits | 1 | 20.61 | <0.001 |
|  | Percentage of women who had four ANC visits | 5 | 8.58 | 0.003 |
|  | Percentage of women who had four ANC visits | 6 | 9.90 | 0.002 |
|  | Percentage of women who had skilled delivery | 1 | 19.61 | <0.001 |
| Upper West | Percentage of women who had four ANC visits | 1 | 24.86 | <0.001 |
|  | Percentage of women who had four ANC visits | 6 | 6.98 | 0.008 |
|  | Percentage of women who had four ANC visits | 7 | 3.96 | 0.047 |
|  | Percentage of women who had skilled delivery | 1 | 20.49 | <0.001 |
